# Assessment of Pathologic Response in Patients With Locally Advanced Rectal Cancer: Implications of the Park et al. Study

**Published:** 2013-11-01

**Authors:** Annie R. Truong, Steven H. Wei

**Affiliations:** From MD Anderson Cancer Center, Houston, Texas

Neoadjuvant chemoradiotherapy is considered the current standard of care for patients with locally advanced rectal cancers. The study by Park et al. (2012) discussed by Wolf and Malatek on page 438 represents the largest single-institution study to date evaluating response-stratified outcomes following chemoradiotherapy and radical resection for patients with clinical stage II or III disease (Park et al., 2012). The findings from the MD Anderson group further validate the growing sentiment that pathologic complete response (pCR) is associated with improved local control and survival. However, questions remain about the practical implications of such data and how having a powerful neoadjuvant treatment response indicator may impact strategies in patient care as well as future research.

## Study Strengths

The study by Park and colleagues provides a new response-stratified oncologic benchmark to which new strategies can be compared. Some obvious strengths of this study include the large sample size (N = 725) spanning a 15-year period, which lends itself to excellent statistical power, contributing to the study’s overall reliability. Since all patients were treated at the same cancer institution, it allowed for uniformity of patient staging, imaging, and laboratory studies. In addition, the timing and administration of appropriate tests, along with their interpretations, were likely performed with the same methodology and standards. Furthermore, the administration of chemotherapy, radiotherapy, and surgery was performed in a consistent manner: All treatments were administered at a single institution, thus ensuring good adherence to treatment guidelines along with quality assurance of those treatments. Furthermore, as it is difficult to standardize how pathologic findings are interpreted by pathologists at different institutions, having all surgical specimens assessed by the same group of pathologists helps ensure consistent quality in the reporting of findings.

**Figure 1:**
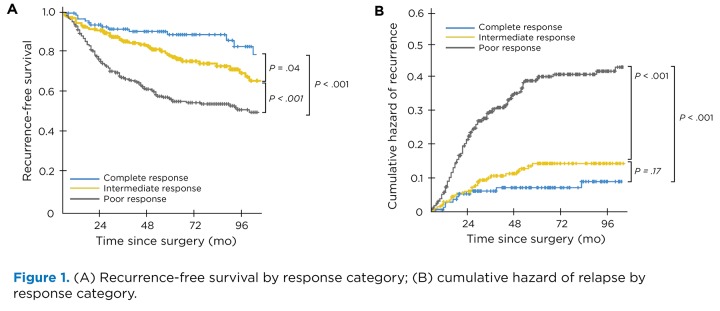
Figure 1. (A) Recurrence-free survival by response category; (B) cumulative hazard of relapse by response category.

Another advantage of the Park et al. study is the evaluation of various levels of tumor response on disease recurrence and survival, not just those with pCR. The ability to stratify treatment response following neoadjuvant therapy provides clinicians and patients with important prognostic indicators for both good and poor responders. The response outcomes of each of the tumor-response variables were well described in the study, and the survival curves for each response group were prominently depicted using Kaplan-Meier (KM) analyses (Figure 1).

## Use of Kaplan-Meier CurVes

In the Park et al. study, all survival analyses were conducted using the KM method, a widely popular technique used to estimate survival in biomedical research. This method provides an estimate of the survival function based on a series of time intervals, each containing one observed death (e.g., "dot" or vertical line) at the start of each interval. A plot of the KM estimate results in a survival curve in which the estimated survival probabilities are constant between adjacent death times and only decrease to a lower level at each death event (Kaplan & Meier, 1958).

An important advantage of the KM method is that it can take into account "censored" data, or losses from the sample of which only partial information is known before the final outcome is observed. Another benefit of the KM method is that it allows graphic representation of multiple treatment groups, comparing the efficacy of each group as it relates to survival. In addition, the KM method is particularly effective in estimating survival when the sample size is large, allowing the graph to approach the true survival function.

However, a major limitation of the KM method also involves the inclusion of censored data, or missing values from incomplete data, which can lead to error. As time progresses, the sample size gets smaller, and the survival curve becomes less accurate. The area where the plot ends, to the right of the graph, depicts the greatest level of uncertainty. Additionally, competing risks, such as death from other causes, are not well represented by the survival curves.

In the Park et al. study, KM analysis was the ideal method for illustrating response-stratified outcomes from three main groups. Having both a large sample size (N = 725) and a relatively long median follow-up duration (65 months) contributed to a more accurate model of survival outcomes. Additionally, collecting data from a single institution eliminates interinstitutional biases and variations in treatment practices that may occur if pooling data from multiple institutions. In the study, the KM method was used to evaluate the primary endpoint of 5-year recurrence-free survival (RFS), but secondary outcomes were also measured: 5-year overall survival (OS), distant metastasis (DM) rates, and local recurrence (LR) rates. The survival differences between the three tumor response groups (complete response [CR], intermediate response [IR], and partial response [PR]) were compared by using the log-rank tests, a popular method of comparing survival curves for the entire cohort based on the same assumptions on censoring and survival probabilities as the KM method. Because the log-rank test is purely a test of significance between curves, it cannot provide an estimate of the size of the difference between groups or explore the effects of significant clinical factors (Bland & Altman, 2004).

**Figure 2:**
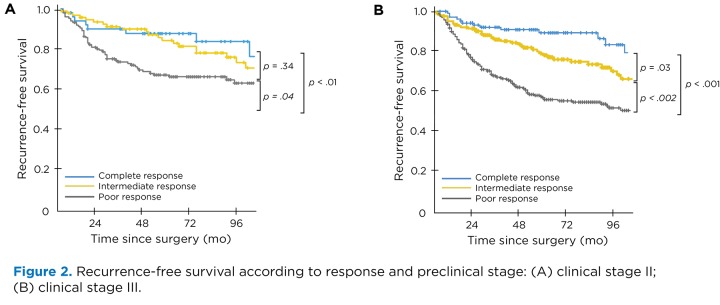
Figure 2. Recurrence-free survival according to response and preclinical stage: (A) clinical stage II; (B) clinical stage III.

The Cox regression model is another survival analysis technique that is used to investigate the effect of several independent variables over time. Using multivariate analysis, the RFS was noted to be strongly associated with treatment response to chemoradiotherapy and only weakly related to pretreatment clinical stage (Figure 2). When compared with the CR group, the PR group was strongly related to an increased risk of recurrence (hazard ratio [HR], 3.01; 95% confidence interval [CI] = 1.75–5.16).

## Summary of Study Findings

In all, 725 patients were treated with neoadjuvant chemoradiotherapy and were classified by tumor response: complete (131 patients; 18.1%), intermediate (211; 29.0%), and poor (384; 53.0%). Age, sex, clinical nodal (N) stage, and tumor location were not related to tumor response. Tumor response (CR vs. IR vs. PR) was associated with 5-year OS (93.4% vs. 87.0% vs. 77.3%), RFS (90.5% vs. 78.7% vs. 58.5%; * p* < .001), 5-year DM rates (7.0% vs. 10.1% vs. 26.5%; * p* < .001), and 5-year LR only rates (0% vs. 1.4% vs. 4.4%;* p* = .002). For the entire cohort, the 5-year OS and RFS rates were 82.9% and 70.1% (Park et al., 2012).

The results from the Park et al. study concluded that oncologic outcomes after preoperative chemoradiotherapy followed by radical resection in locally advanced rectal cancer patients correlate with disease treatment response. The degree of the response rates was based more on pathologic evaluation and less on clinical information. Patients with CR following radical resections had low rates of distant disease recurrence without isolated local recurrence. The patients who developed IR had improved rates of distant disease recurrence and local recurrence when compared with patients with PR.

## Study Limitations

The main potential limitation of this study is the inherent weakness of collecting data from a retrospective review during a 15-year study period, which may include variations in diagnosis and treatment as well as possible errors in data collection. For example, the quantitative radial margin distance was not consistently reported in some patients who were treated earlier. Furthermore, only medically fit patients received adjuvant therapy (84.3%), and those who received chemotherapy may have had different chemotherapy regiments such as single-agent fluorouracil (5-FU), oral capecitabine, or other combinations including oxaliplatin. Since local recurrence was generally uncommon for all three treatment groups and the pattern of failure was mostly systemic, especially among those in the PR group, it may have been useful to stratify further those patients who went on to receive adjuvant chemotherapy. Overall, the evaluation of the impact of adjuvant therapy on survival outcomes was not the focus of this study, but it should be considered for future investigation.

## Practical Implications

Pathologic response serves as an early surrogate marker or predictive indicator for long-term oncologic outcomes, such as OS and RFS. This response-stratified rate is a powerful prognostic indicator for physicians and advanced practitioners (APs) to help counsel patients about various adjuvant treatment options. Physicians and APs play an important role in discussing the risks and benefits of treatment, how these treatment modalities may influence long-term survival, as well as quality-of- life concerns. Some examples of possible risks of treatment include chemotherapy- and radiation-related toxicities, potential surgical complications, and lifestyle changes related to radical surgery. Patients who are well educated about their treatment choices can better understand their potential therapeutic options and make informed decisions to help improve their overall quality of life. For example, patients with pCR following radical resection may decide to forgo any adjuvant chemotherapy and opt for continued surveillance or observation.

Also, some patients may decide to extend the interval of surveillance visits due to being "good responders." As discussed in the preceding article by Wolf and Malatek, the "wait-and-see" approach and organ-preserving strategies have been the subject of much controversy but continue to remain practical options worth discussing among patients with favorable CR. Alternatively, for patients with IR or PR following neoadjuvant therapy, the physician and the AP play key roles in educating patients on the importance and utility of adjuvant systemic therapy, possibly guiding these patients toward treatment intensification based on pathologic data.

## "Wait-and-See" Policy

In the Park et al. study, 84.3% of patients received postoperative adjuvant chemotherapy. Some common factors leading to patients not receiving treatment include major postoperative complications or poor performance status following surgery. For patients with a pCR, it may be feasible for them to forgo additional adjuvant chemotherapy and continue with oncologic surveillance studies and clinic visits only. Approximately 15% to 20% of patients who undergo neoadjuvant chemotherapy for locally advanced rectal cancers experience pCR after radical resection (Maas et al., 2011). Habr-Gama and colleagues (2004) investigated whether it was possible to clinically predict those who might achieve pCR by assessing them during the preoperative period for possible clinical complete response (cCR). It was thought that patients with cCR may benefit from a wait-and-see policy and may have comparable long-term benefits compared with those patients with pCR following radical surgery (Habr-Gama et al., 2004).

In the more recent study by Maas et al. (2011), 21 patients in the control group with pCR after chemoradiotherapy and TME were compared with 21 patients with cCR who adopted the wait-and-see policy. Of the 21 patients in the wait-and-see group, one developed a small endoluminal recurrence, and the rest were disease-free. For the wait-and-see group, the cumulative probability for 2-year disease-free survival (DFS) was 89% and that for OS was 91%. The control group had a cumulative probability for 2-year DFS of 93% and that for OS was 91%. Two patients from the control group died: One died from surgical complications, and the other died from metastatic disease. This study concluded that cumulative probabilities of DFS and OS from both groups were not significantly different, and there appeared to be a group of patients with cCR who may be able to avoid radical resections.

One could argue that patients with cCR may consider an "organ-preserving" strategy or the wait-and-see approach, especially those expressing a strong preference for avoiding major abdominal and pelvic surgery, including ileostomy/colostomy, by proceeding with observation or local excision, such as transanal excision. As a result, physicians and APs need to consider several factors, including pathologic response, the patient’s medical comorbidities, and the remaining risk of 9% lymph node involvement in ypT0 (where yp indicates a posttreament pathologic designation) groups (Hughes et al., 2006), when counseling patients on treatment strategy. Many physicians are reluctant to treat patients without surgery, especially since there is a lack of sufficient modalities to measure clinical response rates (e.g., cCR) prior to surgery.

Some modalities used to measure clinical response include digital rectal exam; endoscopic exams (with biopsy); MRI to rule out local recurrences; CT of the chest, abdomen, and pelvis to evaluate for metastatic disease; and carcinoembryonic antigen. However, the most reliable current modality to measure response rate remains the pathologic response, where patients undergo standard resection with total mesorectal excision and the specimen is evaluated by a GI pathologist. To date, there continues to be no good surrogate method for identifying patients with pCR aside from standard resection. The findings for using the wait-and-see policy in complete responder groups are encouraging, but it needs to be subject to a further investigation, including possible randomized study.

## The Role of Adjuvant Therapy

Evaluation of the impact on survival outcomes from adjuvant chemotherapy was not thoroughly analyzed in the 2012 study by Park et al. In addition, neoadjuvant treatment response was not found to be associated with whether or not a patient received adjuvant treatment. This study demonstrated that neoadjuvant treatment response not only provided an indicator for prognosis, but also served as a potential indicator for the use of certain chemotherapy agents in the adjuvant setting. For example, patients with pCR are considered good responders and may benefit from the same chemotherapy agent used during neoadjuvant chemoradiotherapy. Furthermore, patients with IR or PR may need alternative or intensified therapeutic options to reduce the risk of systemic failure. Unfortunately, there are limited data on adjuvant chemotherapeutic treatments for rectal cancer. The only current guidelines available are trials of adjuvant chemotherapy for colon cancer.

As described by Collette and colleagues, the European Organisation for Research and Treatment of Cancer (EORTC) 22921 trial evaluated patients following curative resection for cT3-T4 rectal cancers after preoperative chemoradiotherapy and questioned whether patients benefited from adjuvant 5-FU–based chemotherapy (Collette et al., 2007). The results revealed no statistically significant impact of adjuvant chemotherapy on DFS for the whole group (* p* > .5) and only ypT0-T2 patients benefiting from adjuvant chemotherapy. The DFS rate was 65.6% with chemotherapy and 76.7% without chemotherapy. For patients without downstaging (ypT3-T4), there was no significant benefit of adjuvant chemotherapy. This group had a 5-year DFS rate of 48.9% without chemotherapy and 45.1% with adjuvant chemotherapy. Overall, the EORTC trial 22921 did not demonstrate improvements in DFS or OS for patients who received adjuvant chemotherapy.

Future research should continue to investigate the role of adjuvant chemotherapy, especially among good responders with pCR, who may receive similar oncologic outcomes after a wait-and- see approach, without the potential toxicities related to additional therapy. Furthermore, future studies may need to examine newer and/or intensified chemotherapy regimens, including possible targeted therapies, for patients with IR or PR who are considered poor responders. Additional advances continue to emerge, with a focus on gene expression profiles of the primary tumor, pathologic assessment of tumors, radiotherapy modalities, and advances in radiographic imaging. These advances will hopefully improve tumor response, OS, and DFS.

## Conclusion

Neoadjuvant chemoradiotherapy followed by radical resection remains the current standard of care for patients with locally advanced rectal cancer. The current study by Park et al. provides response-stratified oncologic benchmarks for comparison of novel treatment strategies. With the advances in treatment options, along with the complexity of patient decision-making, the role of the AP and physician team becomes even more crucial in helping to properly educate and inform patients of their disease process and predictive outcomes, as well as the current standard of care. Patients who are educated about their treatment choices can better make informed decisions, seek treatments that would in turn improve overall quality of life, and possibly even improve survival. For patients who are survivors but are burdened by long-term side effects of treatment, any advancement in treatment strategies that could alter and improve the length of therapy, as well as reduce the morbidities associated with treatments, would be a huge benefit across all subpopulations of rectal cancer survivors.
